# When does hearing laughter draw attention to happy faces? Task relevance determines the influence of a crossmodal affective context on emotional attention

**DOI:** 10.3389/fnhum.2012.00294

**Published:** 2012-10-26

**Authors:** Pieter Van Dessel, Julia Vogt

**Affiliations:** ^1^Department of Experimental-Clinical and Health Psychology, Ghent UniversityGhent, Belgium; ^2^Booth School of Business, University of ChicagoChicago, IL, USA

**Keywords:** affective context, crossmodality, emotional attention, task relevance, attentional bias

## Abstract

Prior evidence has shown that a person's affective context influences attention to emotional stimuli. The present study investigated whether a crossmodal affective context that is induced by remembering an emotional sound modulates attention to visual emotional stimuli. One group of participants had to remember a positive, negative, or neutral sound during each trial of a dot probe paradigm. A second group of participants also had to encode the valence of the sound. The results revealed that attention was preferentially deployed to stimuli that were emotionally congruent to the affective context. However, this effect was only evident when participants had to encode the valence of the affective context. These findings suggest that a crossmodal affective context modulates the deployment of attention to emotional stimuli provided that the affective connotation of the context is task-relevant.

## Introduction

Numerous studies have shown that attention is preferentially deployed to emotional stimuli (Yiend, 2010) and especially to negative events (Rozin and Royzman, 2001). Many researchers assume that this negativity bias has an evolutionary benefit since the detection of dangers is relevant to survival (Öhman et al., 2001). Recently, however, it has been discussed whether a negativity bias is adaptive at all times (Rothermund et al., 2008). For instance, strong attentional biases to negative events are related to deficits in psychological adaptation (Gotlib et al., 2005). Some researchers have therefore suggested that the deployment of attention needs to be flexible in order to be adaptive (Brandtstädter and Rothermund, 2002).

In line with this reasoning, Smith et al. (2006) have proposed that the accessibility of positive or negative information in memory determines whether positive or negative emotional stimuli receive preferred attention. According to their reasoning, highly accessible negative information signals to watch out for dangers, thereby tuning attention to negative events. In contrast, the accessibility of positive information indicates safety, permitting attention to be deployed to positive, potentially rewarding information. Indeed, when participants had to indicate the valence of predominantly positive or negative pictures, they preferentially deployed attention, as indexed by the P1 component of event-related brain potentials (ERPs), to pictures that matched the valence of the majority of the presented pictures. Relatedly, Becker and Leinenger (2011) have found an attentional bias toward mood-congruent stimuli. Moreover, Grecucci et al. (2010) have demonstrated that holding emotional words in memory directs attention toward emotionally congruent faces.

The present research aims to extend these findings by investigating whether inducing an affective context in one modality affects the allocation of attention to affectively congruent and incongruent stimuli presented in a different modality. For instance, when a person hears laughter, is attention biased to visually presented positive stimuli such as happy faces? In real life, people are constantly presented with information in different modalities and people appear to integrate this information (Spence, 2007). Moreover, previous research has shown that visual and auditory emotional stimuli modulate the attentional capture of an acoustic probe, as indexed by ERP-component P3, in a similar way (Keil et al., 2007). In addition, Brosch et al. (2008) have shown that emotional sounds bias the deployment of visual attention to neutral events that appear in the same spatial location. However, these findings do not allow any conclusions on whether auditory emotional information biases attention toward certain classes of information such as emotionally congruent visual input. Observing a general, modality-independent influence of an affective context on emotional attention would suggest that contextual influences on emotional attention are much more powerful and general than when they would be limited to an exact overlap of the modality of affective context and emotional input (cf. Scherer and Larsen, 2011).

To address this issue, we investigated whether remembering an emotional sound modulates the attentional bias toward positive and negative visual stimuli. Moreover, we included a condition in which we examined whether the influence of an auditory context is dependent on the task relevance of the affective connotation of the context. Recent evidence has suggested that affective information needs to be task-relevant in order to observe an attentional bias toward emotional stimuli (e.g., Hahn and Gronlund, 2007; Van Dillen et al., 2011). If this is also true for contextual influences, then the impact of an affective context should depend on the relevance of the affective connotation for the task at hand.

We used a standard dot probe task (MacLeod et al., 1986) to examine the orienting of attention. In this task, one positive or negative picture and one neutral picture were simultaneously presented at two different locations on the screen, immediately followed by a target. If individuals selectively orient to a certain type of picture, responses should be faster to targets at the location previously occupied by that picture. Before each trial of the dot probe task, we induced an affective context by presenting a neutral, positive, or negative sound that participants had to remember during the dot probe task trial. According to prominent models of attention (Folk et al., 1992; Folk and Remington, 1999), attentional capture is contingent upon top–down settings and holding information in working memory therefore biases attention toward matching information (Downing, 2000; Soto et al., 2007). After each trial of the dot probe task, we presented a second sound that could match the first sound or not. In experimental condition one, participants had to indicate whether the sound was identical or different to the first sound. In condition two, they had to indicate whether the sounds were identical or the valence of the sounds was the same. By this, participants had to encode both the sound and its affective connotation.

## Materials and methods

### Participants

Sixty native Dutch-speaking volunteers (30 women) took part in the experiment. Thirty participants were assigned to experimental condition one and thirty different participants were assigned to condition two. Participants had normal or corrected-to-normal vision. Participants were naive as to the purpose of the experiment and gave written consent prior to participating in this study.

### Apparatus and materials

#### Auditory stimuli

Forty-five sounds were either extracted from a sound database (http://www.findsounds.com) or were recorded for the goal of this study. Sounds consisted of screaming, mumbling, or laughing for a duration of 1500 ms and were performed by women. We restricted both sound and visual stimuli to women to avoid gender influences. We selected eight sounds for each sound category (positive, neutral, or negative), based on a pretest in which 47 participants provided ratings of valence and arousal on a seven-point Likert scale ranging from “1” (not at all pleasant/arousing) to “7” (completely pleasant/arousing). Participants also assessed whether a man or a woman had produced the sound. Three criteria were used for the selection: first, all participants had to rate the producer of the sound as female. Second, the valence ratings had to be significantly different between all sound categories (positive: *M* = 5.16, *SD* = 0.23; neutral: *M* = 3.63, *SD* = 0.14; negative: *M* = 1.54, *SD* = 0.21), *p*s < 0.001. Finally, we sought to match the arousal level of positive sounds (*M* = 4.63, *SD* = 0.24) as closely as possible to the arousal level of negative sounds (*M* = 4.96, *SD* = 0.42), *t*_(7)_ = 2.28, *p* = 0.06.

#### Visual stimuli

Twenty-four pictures were obtained from the Karolinska Directed Emotional Faces database (KDEF, Lundqvist et al., 1998). We selected eight pictures for each picture category (positive, neutral, or negative). These pictures depicted a woman's face with either a laughing, neutral, or fearful expression. Selection was based on a validation study by Goeleven et al. (2008) in which participants evaluated all pictures on emotional content and provided ratings for arousal on a nine-point Likert scale ranging from “1” (not at all arousing) to “9” (completely arousing). We selected the pictures on the basis of two criteria. First, the emotional expression of the picture was unambiguously correctly identified (i.e., more than 70% correct identifications) in the study by Goeleven et al. Second, the arousal level of positive pictures (*M* = 3.93, *SD* = 0.30) matched that of negative pictures (*M* = 3.83, *SD* = 0.32), *t*_(7)_ = 0.86, *p* = 0.42.

### Procedure

#### Experimental condition 1

The experiment was programmed and presented using the INQUISIT Millisecond Software package (Inquisit 3.0, 2011) on an Asus Barebone Computer. The participants sat at a viewing distance of 54 cm from a 17-in. CRT monitor. On each trial, participants had to perform a combination of an auditory working memory task with a visual dot probe paradigm. First, an emotional sound was presented over a headphone and participants were asked to remember this sound. Then, participants saw two cue pictures and had to respond to a subsequently presented visual target in the dot probe task. Immediately afterward, participants were tested on their recollection of the remembered sound by judging whether a second sound presented at this point was identical or different to the first sound.

**Figure 1:**
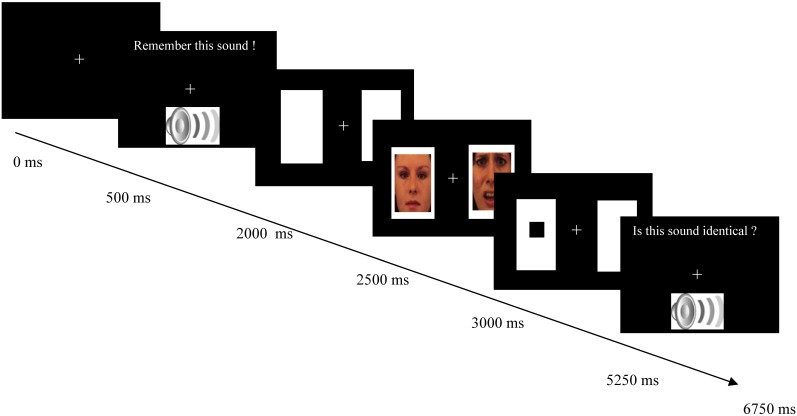
**Schematic overview of an example trial of the combined auditory working memory task and the visual dot probe task.** The first two boxes displays the onset of the working memory task in which an emotional auditory stimulus was presented that had to be remembered during the dot probe task. The next three boxes depict the dot probe task in which the presentation of two cues was followed by a probe (black square) that had to be localized. The cues consisted of an emotional picture, which was either positive or negative, and a neutral picture. The last box displays the second part of the working memory task in which a second auditory stimulus was presented. Participants had to evaluate whether this sound was identical to the first sound. In experimental condition 2, a different message appeared together with the second sound, namely “Is this sound similar in emotional value?” on half of the trials. On those trials participants had to evaluate whether the second sound was similar to the first sound in valence, which was either neutral, negative, or positive.

As can be seen in Figure 1, each trial started with a white fixation cross (0.6 × 0.6° visual angle) presented against a black background in the middle of the screen. After 500 ms an emotional sound appeared along with the message “Remember this sound!” on the screen. Hereafter, two white rectangles (14.4 × 13.5° visual angle) were presented, one to the left and one to the right of the fixation cross. The middle of each of these two peripheral rectangles was 8.8° visual angle from fixation. After 500 ms, two pictures (12.1 × 11.3° visual angle) were presented in the rectangles for 500 ms. After picture offset, a target appeared. The target consisted of a black square (1.1 × 1.1° visual angle) presented in the center of one of the two rectangles. Participants had to respond by pressing one of two keys (target left: “4”; target right: “5”) with the index and middle finger of their right hand on the numeric pad of an AZERTY keyboard. After a response was registered or 1500 ms had elapsed since target onset, a fixation screen was presented for 750 ms. Then, a second emotional sound was presented together with the question “Is this sound identical?” Participants had to respond by pressing one of two keys (same sound: “s”; different sound: “d”) with the index and middle finger of their left hand. Feedback on the correctness of their response was displayed for 500 ms after their response had been registered. The next trial started after 500 ms.

The experiment consisted of 180 trials, 60 trials for each of the three sound valence categories: neutral, positive, or negative. The second sound was identical to the first sound in 50% of all trials. In each trial of the dot probe task a neutral picture was presented with an emotional picture. The emotional picture was positive in half of the trials and negative in all other trials. Half of all trials were *emotionally valid* trials, in which the target appeared on the same side as the emotional picture. In *emotionally invalid* trials the target appeared on the same side as the neutral picture. The order of trials was determined randomly and for each participant separately. Participants in condition 1 performed 10 practice trials and participants in condition 2 performed 12 practice trials.

#### Experimental condition 2

The procedure for participants in condition 2 was identical to condition 1, except that the memory task changed. During the instructions, participants were informed that sounds would be positive, neutral, or negative in emotional value. In 50% of the trials, participants had to indicate whether the second sound matched the first sound in affective connotation. The message that appeared on the screen was “Is this sound similar in emotional value?”. Participants had to respond to this question in the same way as in condition 1 (i.e., same emotional value: “s”; different emotional value: “d”). To prevent those participants would only memorize the valence but not the sound, participants indicated whether the sounds were identical or different in the other half of the trials. By this, participants did not know which judgment they had to perform until the second sound was presented.

## Results

### Data preparation

Trials with errors on the dot probe task were removed (experimental condition 1: 1.13%, condition 2: 3.90%). Participants made errors on the working memory task in 4.21% of the trials in condition 1 and in 8.00% of the trials in condition 2. Dot probe trials followed by an erroneous response in the working memory task were not included in the analyses. Data from one participant in the first condition were removed because she gave an incorrect response in over 25% of dot probe task trials. In line with Vogt et al. (2011a), dot probe reaction times (RTs) shorter than 150 ms or larger than three standard deviations above the individual mean were discarded as outliers (condition 1: 3.86%; condition 2: 0.01%).

### Overall effects

We performed a 3 (sound valence: positive, neutral, negative) × 2 (picture valence: positive, negative) × 2 (emotional cue validity: valid, invalid) repeated measures analysis of variance (ANOVA) on the RTs of the dot probe task with experimental condition as between-subject factor. There was a significant effect of emotional cue validity, *F*_(1, 57)_ = 4.80, *p* = 0.014, η^2^_p_ = 0.08. Responses were faster on trials in which the target appeared at the location of the emotional picture (*M* = 434 ms, *SD* = 15 ms) than at the location of the neutral picture (*M* = 442 ms, *SD* = 14 ms). Importantly, the main effect of experimental condition did not reach significance, *F*_(1, 57)_ = 0.50, *p* = 0.48, η^2^_p_ = 0.01, meaning that reaction times in experimental condition 1 were not different from condition 2 (condition 1: *M* = 428 ms, *SD* = 20 ms; condition 2: *M* = 448 ms, *SD* = 20 ms). The main effects of sound valence and picture valence did not reach significance either, *F*s < 2.50, *p*s > 0.11.

The interaction between picture valence and emotional cue validity was significant, *F*_(1, 57)_ = 6.50, *p* = 0.013, η^2^_p_ = 0.10. Responses were faster on trials where the location of the negative picture was valid (*M* = 432 ms, *SD* = 15 ms) compared to invalid (*M* = 445 ms, *SD* = 14 ms), *t*_(58)_ = 3.32, *p* = 0.002. This was not the case for positive pictures (valid: *M* = 435 ms, *SD* = 15 ms; invalid: *M* = 440 ms, *SD* = 14 ms), *t*_(58)_ = 1.00, *p* = 0.32. The analyses also revealed a significant interaction between emotional cue validity and experimental condition, *F*_(1, 57)_ = 8.21, *p* = 0.006, η^2^_p_ = 0.13. Responses were faster in condition 2 on emotionally valid trials (*M* = 438 ms, *SD* = 26 ms) compared to emotionally invalid trials (*M* = 458 ms, *SD* = 23 ms), *t*_(29)_ = 2.73, *p* = 0.011. This difference was not significant in condition 1 (valid: *M* = 429 ms, *SD* = 21 ms; invalid: *M* = 427 ms, *SD* = 19 ms), *t*_(28)_ = 0.98, *p* = 0.33.

Crucially, the interaction between sound valence, picture valence, and emotional cue validity was significant, *F*_(2, 114)_ = 6.91, *p* = 0.001, η^2^_p_ = 0.11. This interaction was qualified by the four-way interaction effect between sound valence, picture valence, emotional cue validity, and experimental condition, *F*_(2, 114)_ = 4.00, *p* = 0.021, η^2^_p_ = 0.07. None of the other two- or three-way interactions reached significance, *F*s < 1.15, *p*s > 0.31.

**Figure 2:**
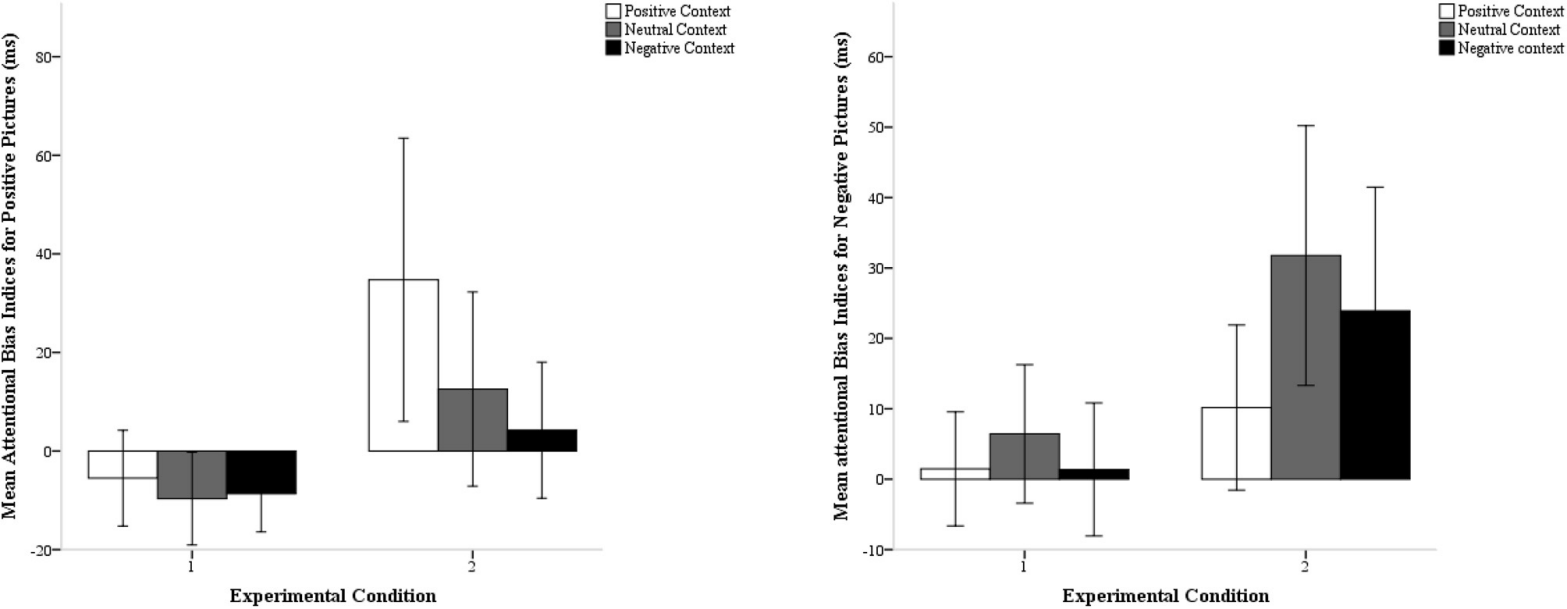
**Mean attentional bias indices for positive pictures and negative pictures as a function of sound valence (positive, neutral or negative) in both experimental conditions**. Error bars represent 95% confidence intervals.

To further explore the latter effect, we conducted separate ANOVAs for each condition. The three-way interaction between sound valence, picture valence, and emotional cue validity was significant in the second condition, *F*_(2, 58)_ = 7.52, *p* = 0.001, η^2^_p_ = 0.21, but not in the first, *F*_(2, 56)_ = 0.62, *p* = 0.54, η^2^_p_ = 0.02. We then calculated indices for attentional biases by subtracting RTs on emotionally valid trials from RTs on emotionally invalid trials for positive and negative pictures separately (Vogt et al., 2010) (see Table 1; Figure 2). In condition 1, significant attentional biases to either positive or negative pictures were not revealed, *t*s < 1.40, *p*s > 0.19. In contrast, in condition 2, participants displayed a significant attentional bias to positive pictures when they remembered positive sounds (*M* = 35 ms, *SD* = 76 ms), *t*_(29)_ = 2.79, *p* = 0.009, and to negative pictures when they remembered negative sounds (*M* = 24 ms, *SD* = 47 ms), *t*_(29)_ = 2.47, *p* = 0.019. The negativity bias was also significant when participants remembered neutral sounds (*M* = 32 ms, *SD* = 49 ms), *t*_(29)_ = 3.52, *p* = 0.001. Planned comparisons revealed that the positivity bias was significantly larger in a positive context (*M* = 35 ms, *SD* = 77 ms) than in a negative (*M* = 4 ms, *SD* = 37 ms) or neutral context (*M* = 13 ms, *SD* = 53 ms), *t*s > 2.31, *ps* < 0.03. The negativity bias was significantly larger in a negative context (*M* = 24 ms, *SD* = 47 ms) than in a positive context (*M* = 10 ms, *SD* = 31 ms), *t*_(29)_ = 2.40, *p* = 0.023, but not significantly larger than in a neutral context (*M* = 32 ms, *SD* = 49 ms), *t*_(29)_ = −0.87, *p* = 0.39.

**Table 1:** **Mean attentional bias indices for positive and negative pictures and standard deviations (in ms) as a function of emotional sound valence in condition 1 and 2**.

	**Affective context**					
	**Positive sound**		**Neutral sound**		**Negative sound**	
	***M***	***SD***	***M***	***SD***	***M***	***SD***
**CONDITION 1**						
Positive pictures	−6	26	−10	25	−9	20
Negative pictures	1	21	6	26	1	25
**CONDITION 2**						
Positive pictures	35^*^	76	13	53	4	37
Negative pictures	10	31	32^*^	49	24^*^	47

Note: Attentional bias indices for positive and negative pictures were calculated by subtracting RTs on emotionally valid trials from emotionally invalid trials

* p < 0.05.

## Discussion

The aim of this study was to examine whether remembering emotional sounds modulates the allocation of spatial attention to emotional pictures. We found that an affective auditory context modulated visual attention, when the task required participants to encode the valence of the affective context. In this case, more attention was allocated to pictures that were emotionally congruent to the remembered sound. These results add to recent findings on the influence of affective contexts on attention (Smith et al., 2006; Grecucci et al., 2010).

Importantly, in our study, the influence of an affective context extended to another modality. The auditory affective context modulated attention to positive or negative emotional stimuli in the visual modality. This suggests that the influence of an affective context on emotional attention involves representations that are modality-independent and abstract rather than modality-specific (Peelen et al., 2010). In a neuroimaging study by Klasen et al. (2011), the ventral Posterior Cingulate Cortex (vPCC) has been suggested as a neurological basis for supramodal representations of emotion. These supramodal representations of emotion information would be independent from low-level sensory features and help to determine the relevance of incoming information, through links with the Anterior Cingulate Cortex (ACC) (Vogt, 2005). Alternatively, however, the present results might also be compatible with an embodiment view on the representation of emotional categories. Horstmann 2010; (Horstmann and Ansorge, 2011) has argued that emotional categories are represented as *multimodal* sensory-motor representations. Therefore, activating an emotional category in one modality causes the activation of information belonging to this category in other modalities. However, the fact that emotional sounds do only bias attention to matching visual input when the emotional value had to be encoded in an abstract way (i.e., by encoding its valence) suggests that an abstract representation of the emotional information is crucial in order to find these effects on the attentional level. Future evidence is needed to examine the processes underlying crossmodal emotional effects.

Moreover, previous studies (e.g., Smith et al., 2006) only investigated the effects of an affective context by exposing one group of participants to a positive context and another group to a negative context. Our data provide evidence that an affective context influences attention on a trial-by-trial basis. This shows that context effects do not require enduring and rather static mood manipulations (e.g., Becker and Leinenger, 2011), but that attention can be influenced flexibly by the short-term availability of emotional information in memory.

Notably, the affective context did not influence attention when participants did not encode the valence of it. In contrast to previous studies in which participants performed a dot-probe task with emotional cues (e.g., Mogg and Bradley, 1998; Yiend, 2010), participants did not show any bias to emotional stimuli in this condition. However, participants simultaneously had to perform a second task in our study. Previous studies have shown that the processing of emotional stimuli is impaired when another, non-affective task demands cognitive control (e.g., Hahn and Gronlund, 2007; Van Dillen and Koole, 2009). Importantly, the results of this study also show that the affective connotation of the affective context has to be encoded in order to find an influence of the affective context. Interestingly, in the study by Smith et al. (2006), participants had to categorize the emotional pictures that were used for inducing an affective context in terms of emotional valence (i.e., whether they were positive or negative). These results add to findings suggesting that attentional biases to emotional events are driven by the relevance of emotional information for participant's current goals or tasks (e.g., Hahn and Gronlund, 2007). For instance, Vogt et al. (2011b) found that experiencing disgust is accompanied by an attentional bias to disgusting pictures, but also by a bias to pictures representing cleanliness, suggesting that the goal to alleviate an aversive emotion drives emotional attention in aversive emotional states. These findings propose that the influence of emotion on cognition is not automatic in the sense of goal-independent and stimulus-driven as often assumed (e.g., Öhman et al., 2001). In contrast, they corroborate the idea that representations in memory which are determined by the individual's current goals and tasks guide emotional attention (Grecucci et al., 2010; Pessoa and Adolphs, 2010; Vogt et al., 2012; see also Folk et al., 1992).

Four potential limitations of the study need to be addressed. First, we induced a crossmodal affective context by presenting auditory stimuli and measured attentional allocation to visual emotional stimuli. Future research should address whether our findings generalize to other combinations of sensory modalities (e.g., the influence of a visual affective context on attention to auditory or multimodal emotional stimuli). Second, as neutral pictures were presented twice as often compared to positive or negative pictures, the general attentional bias to emotional events can be interpreted as evidence for enhanced attention toward novel stimulus classes (Yantis and Jonides, 1984). However, this cannot explain why an affective context modulates attention toward emotionally congruent stimuli. Third, in line with prior studies using dot probe paradigms and emotional cues we implemented a cue exposure time of 500 ms (e.g., Bar-Haim et al., 2007). Therefore, our results do not allow conclusions on the fast and early allocation of attention. Importantly, we might therefore have measured disengagement-related processes rather than attentional engagement. Moreover, with an exposure time of 500 ms, we cannot exclude possible influences of strategic processes on attention. However, we assume that the use of specific strategies on attention to the emotional cues was limited because emotional cues predicted the correct location of the target in only half of the trials. Fourth, our results revealed that participants preferentially attended to negative stimuli in a neutral context. Though this observation is in line with previous research suggesting that attention is generally biased toward negative stimuli (Baumeister et al., 2001), it could suggest that contextual influences are not the sole determinant of attentional allocation to emotional stimuli. However, we cannot exclude that participants experienced neutral events and negative facial expressions as emotionally congruent. Future research should further examine how both characteristics of emotional stimuli and the affective context distinctively contribute to emotional attention.

In sum, the present study suggests that attention to emotional stimuli is influenced by affective contexts provided that the emotional value of this context is task-relevant. We hope that future research will further explore the relation between emotion and attention across modalities.

### Conflict of interest statement

The authors declare that the research was conducted in the absence of any commercial or financial relationships that could be construed as a potential conflict of interest.
